# Mesothelioma with non-pleural malignancy: a red herring or just an uncommon pairing?

**DOI:** 10.1186/1749-8090-1-39

**Published:** 2006-11-01

**Authors:** Andrew J Drain, Kourosh Saeb-Parsy, Amit K Shah, D Rassl, Andrew J Ritchie

**Affiliations:** 1 Papworth Hospital, Papworth Everard, Cambridge CB3 8RE, UK

## Abstract

Malignant pleural mesothelioma (MPM) is a highly aggressive cancer of the pleura with a well-established male predominance and causative link with asbestos exposure. We report four cases of female patients with MPM referred for palliation of symptoms thought to be due to previous non-pleural malignancy.

With emerging novel treatments for MPM, this article discusses four unusual cases of MPM occurring in the setting of other malignancy, highlights the importance of considering a primary diagnosis of MPM even in patients with other malignancy, and reinforces the benefits of video-assisted surgical biopsy which allows simultaneous diagnosis and treatment.

## Case reports

Four female patients were referred to our institution for definitive treatment of pleural effusion (and one for persistent pneumothorax). All had undergone CT imaging and pleural fluid examination. In all cases the fluid was diagnostic for malignancy but not for MPM. Assuming the pleural effusions to be metastatic from their previous malignancy, they were referred for surgical palliative management.

**Figure 1:**
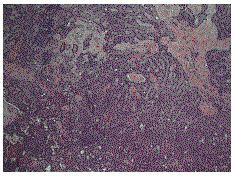
Histology demonstrating epithelioid mesothelioma {x 100}.

**Figure 2:**
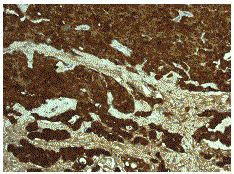
Histology demonstrating epithelioid mesothelioma showing positivity for the immunohistochemical marker, calretinin {x 200}.

The first case was a 59-year-old woman with a history of right-sided adenocarcinoma of the breast for which she underwent mastectomy. After presenting 10 years later with a right-sided pleural effusion and chest wall nodules, she received anastrazole (aromatase inhibitor) and six cycles of docetaxel (taxane) resulting in complete remission of the chest wall disease. The right-sided pleural effusion persisted despite five separate episodes of drainage and so she underwent right-sided video-assisted thoracoscopic surgical (VATS) cytoreductive pleurectomy. The tumour was histologically confirmed to be MPM of the epithelioid type (see Figure 1 and 2). A previous history of asbestos exposure was not confirmed.

The second case involved a 78-year-old woman with a history of right-sided adenocarcinoma of the breast treated with mastectomy and post-operative radiotherapy. She presented 38 years later with a persistent right-sided pleural effusion. Cytological examination of the pleural aspirate showed adenocarcinoma cells consistent with primary breast origin. She was referred for VATS cytoreductive pleurectomy and at operation was found to have infiltration of the pleura with tumour, histologically confirmed to be MPM of the epithelioid type. A previous history of asbestos exposure was not confirmed.

The third case was a 74-year-old woman with a history of endometrial carcinoma for which she underwent a hysterectomy. She presented 20 years later with a persistent right-sided pleural effusion. She underwent VATS cytoreductive pleurectomy and was found to have extensive infiltration of the pleura, the diaphragm and the pericardium with tumour. Histological examination of the tumour confirmed MPM. There was no history of significant exposure to asbestos.

The final case was a 39-year-old woman referred for the surgical treatment of a persistent right-sided pneumothorax and a right sided-pleural effusion. She had a history of nodular sclerosing Hodgkin's lymphoma involving the right lung, treated with mantle radiotherapy 14 years earlier. At operation a bulla was noted in the right lung and the right pleura was found to have a thickened and fibrous appearance, possibly attributable to the previous radiotherapy. The patient underwent VATS bullectomy and cytoreductive pleurectomy. Histological and immunohistochemical examination confirmed the diagnosis of epithelioid MPM. There was no evidence of lymphoma in the lung. A previous history of asbestos exposure was not confirmed.

## Discussion

Malignant pleural mesothelioma (MPM) is an aggressive tumour derived from the lining of the pleural cavity. It frequently presents with dyspnoea and chest pain, and uncommonly with cough, fatigue and weight loss. Occasionally the diagnosis is suspected following a routine chest radiograph, with pleural thickening or a pleural effusion [1,2].

The incidence of MPM is 10–30 per million per year in unselected male populations, and approximately 2 per million per year in female subjects [3]. More than 80 per cent of MPMs develop in individuals with higher than background exposure to asbestos, with an incidence as high as 366/100,000 person-years in heavily exposed workers [4]. The latent interval between first exposure to asbestos and death is very long, with a mean of 41 years and a range of 15–67 years in one series [5]. The use of 'asbestos exposure' as predictor of mesothelioma is however unreliable as many patients are unaware of such exposure or when it occurred. Other potential causative factors of MPMs include the simian virus 40 (SV40), other environmental carcinogens such as erionite, ionising radiation and genetic factors [6]. The role of radiotherapy in inducing malignancy is well described but as yet the link with mesothelioma is unclear.

MPMs have infrequently been reported in patients who had received radiotherapy (with or without chemotherapy) for previous non-pleural malignancy. Examples include the development of MPMs after radiation therapy for breast cancer [7-9] , Hodgkin's lymphoma involving the lung [10-14] and Wilm's tumour with pulmonary metastasis [15]. MPM following chemotherapy alone for breast cancer has also been reported [16]. Isolated cases of concurrence of MPM with breast cancer [7-9] and Hodgkin's lymphoma [10-14] are described.

These cases highlight the importance of the systematic consideration of a diagnosis of MPM in all patients with the typical presentation of a pleural effusion, irrespective of any history of a previous malignancy (patients 1–3) and regardless of previous asbestos exposure. The incorrect assumption that a pleural effusion is caused by the known non-pleural malignancy can be a source of significant delay between presentation and referral. This delay (between 2 and 8 months in the present series) is important in view of the fact that survival after diagnosis of MPMs may be significantly improved as treatment strategies are developed (17,18,19).

Cytological examination of the pleural aspirate in the present series was non-diagnostic. This finding is consistent with the previous reports that routine cytological examination of pleural fluid is generally unreliable for the diagnosis of MPMs, with a sensitivity of only 32% [20]. Similarly, 'blind' percutaneous needle biopsy specimens give a diagnosis in less than 50% of cases [21]. The British Thoracic Society correspondingly recommend that a negative pleural cytology or percutaneous pleural biopsy does not exclude the diagnosis of MPM, and that ultrasound and CT-guided biopsy, and thoracoscopic and surgical biopsy techniques should be used to increase the likelihood of accurate diagnosis [17].

The use of VATS techniques in the management of malignant pleural effusions including MPM is well established [19]. It now has few complications and allows both diagnosis and therapeutic intervention to be achieved at one operative episode.

Three out of four cases in this series received significant doses of radiotherapy as part of the original treatment of their tumours. Previous cases have demonstrated the link between radiotherapy and the development of malignancy with a time frame not dissimilar to that for mesothelioma after asbestos exposure. This and other reports now suggest that the combined exposure to both asbestos and radiotherapy may be synergistic.

## Conclusion

The diagnosis of malignant pleural mesotheliomas should be actively considered in all patients presenting with effusion relating to previous malignancies regardless of asbestos history. As treatment options develop for mesothelioma early and accurate diagnosis is increasingly important and best achieved by guided CT or VATS biopsy since cytology and blind needle aspiration are of low yield and may be misleading in this context. The exposure of asbestos particles to a radiotherapy field carries a theoretical risk of synergy, which requires further study [6].
